# Biomimetic approaches with smart interfaces for bone regeneration

**DOI:** 10.1186/s12929-016-0284-x

**Published:** 2016-11-05

**Authors:** G. S. Sailaja, P. Ramesh, Sajith Vellappally, Sukumaran Anil, H. K. Varma

**Affiliations:** 1 Department of Polymer Science and Rubber Technology, Cochin University of Science and Technology, Cochin, 682 022 India; 2 Biomedical Technology Wing, Sree Chitra Tirunal Institute for Medical Sciences and Technology, Thiruvananthapuram, 695 012 India; 3 Dental Biomaterials Research Chair, College of Applied Medical Sciences, King Saud University, Riyadh, Saudi Arabia; 4 Department of Preventive Dental Sciences, College of Dentistry, Prince Sattam Bin Abdulaziz University, Riyadh, Post Box 153, AIKharj 11942 Saudi Arabia

**Keywords:** Bone regeneration, Biomimetic, Cell-material interaction, Orthopaedic, Smart interface, Bone tissue engineering

## Abstract

A ‘*smart tissue interface*’ is a host tissue-biomaterial interface capable of triggering favourable biochemical events inspired by stimuli responsive mechanisms. In other words, biomaterial surface is instrumental in dictating the interface functionality. This review aims to investigate the fundamental and favourable requirements of a ‘smart tissue interface’ that can positively influence the degree of healing and promote bone tissue regeneration. A biomaterial surface when interacts synergistically with the dynamic extracellular matrix, the healing process become accelerated through development of a smart interface. The interface functionality relies equally on bound functional groups and conjugated molecules belonging to the biomaterial and the biological milieu it interacts with. The essential conditions for such a special biomimetic environment are discussed. We highlight the impending prospects of smart interfaces and trying to relate the design approaches as well as critical factors that determine species-specific functionality with special reference to bone tissue regeneration.

## Background

Biomineralized structures represent one of the classic strategies of evolution success. While their fabulous shapes mesmerized scientists, the complexity associated with them remained as a source of inspiration for engineering several organic-inorganic hybrid structures. Biomineralized structures have been considered unique with respect to their superior hierarchy, species-specific properties like uniform particle size, complex morphology, preferential crystallographic orientation etc. [1]. ‘Biomimetics’, a term coined by Otto Schmitt in 1950s [2, 3] has been recognized as a budding branch of science that explores technological beauty of the nature. The concept of biomimetics has been magnificently explored towards famous applications such as the design of the ‘Eiffel Tower’ by getting inspired from the intriguing trabecular structure of bone (offering it the greatest strength); and the development of novel ‘dirt and water repellent’ paints based on ‘lotus effect’.

‘Biomimetics’ when interacts to biomineralization, leads to incredible inventions in the biomedical field. In fact, the prospects of biomimetically engineered products could be significantly superior to any of its other alternatives. Designing biomimetic constructs requires a greater understanding of nature’s reckoning potential. As a result of this comprehension, the growth of biomimetic approaches has offered valuable insights to many of the present challenges in tissue engineering. Dimasi et al., have shown the unique organization of polymorphs of calcium carbonate in the form of calcite and aragonite in a shell, and how it helps the organism to achieve excellent mechanical properties for its protective covering [4].

The organic-inorganic hybrid materials are multifaceted in their properties and hence offer prolific applications in diverse fields by bridging superior links in a synergistic way. A proper understanding of the frequency of interaction involved in the organic-inorganic interface leads to the recognition of the exceptional potentials of these hybrid materials. Investigations on this topic opened a brighter world of intelligent designing of materials for advanced applications. The organic-inorganic interfaces possess very special properties, and if designed properly; could be explored for addressing many of the presently existing biomedical challenges. The pioneering contributions of Langer and Vacanti [5] paved the way to versatile approaches of bone tissue engineering. The fundamental concept underlying is to design scaffolds with sufficiently interconnected pores of appropriate size to facilitate vascularisation and simultaneously modulating the material surface to hold the potentials to invoke and enhance cellular adhesion and proliferation so that the resulting product could be transformed as a ‘tissue engineered construct’. This further requires knowledge of growth factors and cytokines and their release kinetics [6] and the information regarding local signal transduction that regulate the optimal tissue regeneration pathways [7], growth factor assisted signal transduction [8]. Hence, it could be envisaged that successful orthopaedic tissue regeneration approaches needs to formulate a combinational knowledge consisting of scaffold materials, growth factors and their release kinetics, tissue as well as unit cell properties and more importantly the cross-interaction between these different components in the biological environment.

In addition, orthopaedic regenerative options have recognized bigger challenges due to the inevitable involvement of synchronized interactions of multiple tissues as part of musculoskeletal movement [9]. The degree of clinical translation of a biomaterial directly depends on its biocompatibility and functional integration [10]. It could be seen that in the past few decades there was significant progress in developing such optimally functioning bone graft materials. However, there exists a huge demand for biomaterials capable of integrative repair [11–13]. Combined functioning of growth factors and cells may be thought as a better option for designing functional biomaterials for in vivo tissue engineering and has already been explored for biomimetic design of bone, cartilage, ligament, tendon etc. [14–18]. Recent emphasis is focused on integrated 3D scaffolds and stem cell approaches that simultaneously explore technological advancements and novel design strategies for designing better functional bone grafts [19–22]. A key point which has to be taken care with significant importance is creating a better interface that can invoke desired biological responses and faster healing via osseointegration [23–25].

## Functional interfaces

It is well-known that materials interact with surroundings through their surfaces. The communications involved in such interactions are determined by the material surface properties under specific environments. It is noteworthy to mention that specific interactions (e.g.: ligand-cellular receptor interactions or biomolecule immobilization on a biomaterial surface could be controlled better compared to non-specific interactions (like partial negative charge on the surface of a biomaterial). Hence, considerable efforts have been undertaken by several investigators to impart preferred biofunctionality by introducing biofunctional groups or immobilization of biomolecules onto polymer surface for clinical applications [26–30]. Despite all these efforts, the clinical use of biomimetic materials are in the developmental phase [31]. The surface properties such as surface energy, surface charge and surface roughness play critical roles in determining the cell-material interaction at the interface [32–36]. Surface properties being highly influential in the designing of functional biomaterials, it could be envisaged that an attempt to engineer a successful biomaterial interface and thereby establish a positive interaction in the biological milieu requires a multilateral approach involving knowledge of surface science, material properties and a comprehensive understanding of cellular and molecular biology.

### The organic-inorganic interface

A biomaterial can induce multiplicity of protein mediated cell responses based on its surface characteristics [37–39]. The surface characteristics of a biomaterial thus determine the primary biomolecular response in the biological medium and thereby decide the attachment/orientation of biomolecules on the surface and proliferation/differentiation of cells [33, 40, 41]. An organic-inorganic interface is characterized by its unique chemical sensitivity and inherent species-specific organization potential. The organic-inorganic interface is a highly energy driven regime where the induction of biomimetic mineralization and formation of self-assembled/hierarchical structures could be directed by appropriate designing [42, 43].

### Biofunctionality of the interface and influential factors

‘Biofunctionality’ is a remarkable phenomenon associated with a sequence of favourable events occurring as a result of the interaction between the interface and the host environment. The primary step towards imparting functionality to the interface would be to minimize the unfavourable interactions with biological elements such as proteins and blood cells [44, 45]. There are several basic aspects involved in the fabrication of tailor made biomimetic surfaces with biospecific properties that can elicit a positive interaction between the interface and the host tissue. One of the major approaches followed is through RGD peptide sequences [46–51]. The cell surface possesses diverse types of receptors to facilitate binding with specific proteins present in the extracellular matrix (ECM); which is a complex mixture of glycoproteins and proteoglycans. The RGD peptide sequence mediate the attachment of cells by plasma and ECM proteins, including fibronectin, vitronectin, type I collagen, osteopontin and bone sialoprotein (BSP) [51, 52]. However, the functionality and the cell spreading pattern are directly proportional to the surface density of the peptide [47, 49].

**Fig. 1:**
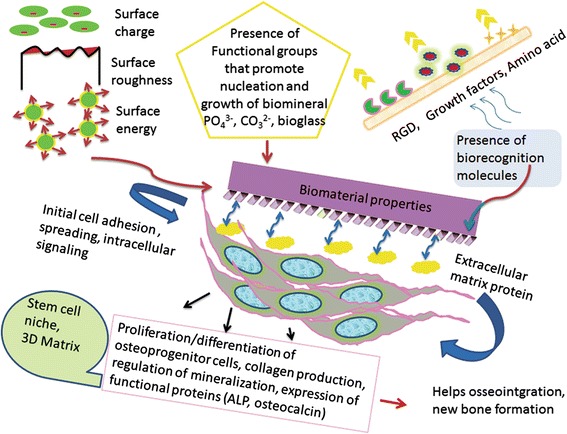
Schematic illustration of the factors involved and molecular cues associated with cell-material interaction

Although there is a significant amount of complexity associated with characterizing the organic-inorganic interface, certain key elements could be considered while fabricating them towards specific applications. Technological design of smart interfaces requires a unique balance of tuning hydrophilic/hydrophobic properties, selection of functional groups or biological molecules to be immobilized and the kinetic control of biomimetic mineralization. Ultimately, this offers exciting opportunities for the researchers to formulate species-specific substrates of their choice. Considering the growth strategy of a biomineral, the functional groups become pivotal [53–55]. With respect to the cell adhesion pattern, the spacer groups/biomolecules attached on the surface play key role [56]. While relating to the stability of the interface in the biological milieu, the cohesive energy of the substrate is an important parameter [57]. The dynamic interaction of the interface with the surrounding medium requires a high accessibility of the surface functional groups. The initial transient interaction between the functional interface and the cellular environment further progresses to an enduring cell-substrate bonding through an appropriate signal transduction pathway. This hypothetical approach when conceived properly could be easily transformed into advanced functional biomaterials. Figure 1 schematically illustrates the factors involved and the molecular cues associated with cell-material interaction.

## The ‘Smart’ interface: from concept to clinical applications

There exists a potential demand for clinically significant bone implants with superior biofunctionality [58–60]. Engineering such functional implants requires a proper understanding of the cell-substrate interaction as well as the fundamental properties of the organic-inorganic interface. Even though several alternatives are presently available for bone augmentation, ranging from bioceramics to polymer-bioceramic composites or bioceramic coated metal components [61–63], attempts towards development of ideal bone graft materials remains as one of the prevailing research topics.

### ‘Smart’ interface: the concept

**Fig. 2:**
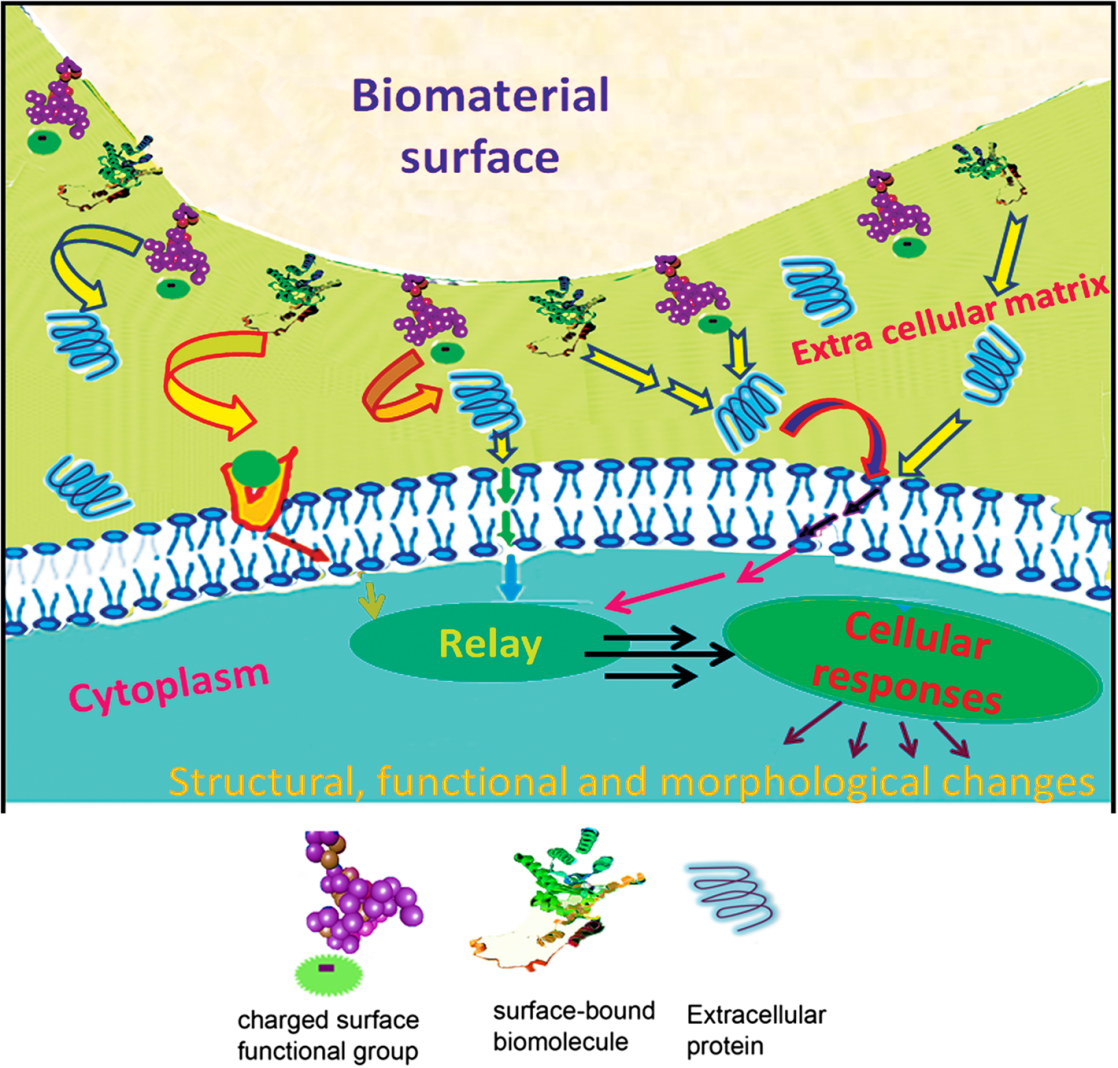
Schematic illustration of a ‘smart ҆ cell-material interface

A ‘smart interface’ could be defined as an interface capable of triggering favourable biochemical events based on stimuli responsive mechanism. A smart interface is capable of responding towards external stimuli and hence organizing by itself by dynamically regulating biological functions. This unprecedented biomimicking property could be imparted to the interface through one or more of intelligent designing approaches like biomolecular immobilization, superior functionalization, stimuli responsive spacer groups etc. [64–66]. The transduction of a bio-recognition event occurs as a result of a biospecific interaction between the functional interface and the cellular environment. Among the various phenomena associated with the cell-material interaction, the first step associated with most of the cell types is adhesion, followed by growth, proliferation and adoption of phenotypic expression [67]. The signature of the mechanism associated with a smart interface with reference to bone tissue regeneration is the ability to elicit a positive interaction between the substrate surface and the cellular environment that simultaneously invokes biomineralization and specific cell binding. The concept of smart interface is schematically illustrated in Fig. 2.

### Signal transduction

It has been understood that cells make direct contact with the surface of an implant through biological signals [33, 68]. The biological signals are generated through primary interaction of the substrate with proteins present in the blood as soon as they come into contact with each other. Moreover, the self-assembly of water molecules on a biomaterial surface has a critical role in determining adsorption/repulsion of proteins or formation of thrombus and thereby the biological response towards it [69]. These extracellular cues are translated into cellular responses by the nucleus to all those cell-material adhesion sites known as focal contacts [70, 71].

The initial cell adherence and spreading pattern are determined by surface characteristics of the material which further directs cellular growth and differentiation as a function of intracellular interactions with extracellular matrix through transmembrane proteins, known as integrins [72–74]. The signal transduction to the cytoskeleton is through bridging focal adhesion proteins i.e., tallin, paxillin and vinculin, which ultimately leads to a positive feedback for the production of ECM by the cell itself [72, 73]. Subsequent activation of focal adhesion (FAK) pathway and mitogen-activated protein kinase (MAPK) cascades further modulate the degree of attachment influenced by the received signals [75]. Integrins also play crucial role in intracellular trafficking and hence, multiple cell functions [76].

### Designing a smart interface for bone tissue regeneration

The worldwide demand for bone grafts is significantly high because of the impending need for functional bone graft materials arising from congenital or acquired bone defects, developmental defects, trauma, presence of tumours etc. [77]. The integration of a bone graft occurs basically through the process of interdigitation [78] a multi-step cascade which involves accumulation of inflammatory cells towards a bone graft as the primary response [79, 80]. This is followed by a chemotaxis of host mesenchymal cells towards the graft site and the primitive host cells differentiate into chondroblasts and osteoblasts. The configuration and chemistry of the biomaterial plays a pivotal role in dictating attachment of the cells and defining its morphology, which finally directs bone formation from the osteoblasts that remodels onto the three dimensional framework of the graft [81, 82].

Bone formation is a result of by collective actions of numerous factors that includes proliferation and differentiation of osteoprogenitor cells, production of collagen, mineralization and its regulation and expression of functional proteins (Alkaline phosphatase and osteocalcin) [83–85]. The type of bone in various part of the skeleton are unique with respect to vascularization, mineral density, porosity and the growth potential and hence the success of long term survival and regeneration of a bone implant is a matter of utmost importance and challenge. Considering the basic difficulties in harvesting the autografts and the lack of true osteoinduction capacities of many of the synthetic materials, there is a huge demand for developing biomimetic materials that can help an increased adherence of osteoblasts onto the biomaterial surface that could promote bone bonding and help osteoinduction [86–88].

An ideal bone graft should provide both the necessary elements for integration and new bone formation cascade and lend structural support during this process. There are many characteristics an ideal bone graft should satisfy. Among these requirements, osseointegration is the most important prerequisite characteristic [89, 90]. The concept of ‘osseointegration’ was presented at the Toronto conference in May 1982 [91]. Even though osseointegration can be defined multiple levels (anatomically, clinically, histologically and ultra structurally), the clinical definition is more relevant in the present context. According to Branemark, ‘osseointegration’ is the direct structural and functional connection between ordered, living bone and the surface of a load carrying implant’. The fracture healing potential of a newly implanted site depends on the well-defined cascade of cellular responses including the foreign body reaction [92]. The remodelling phase primarily depends on the implant-host tissue interaction.

It is possible to formulate strategies for functional designing of interfaces with selective binding sites for bone tissue engineering applications. Biofunctional substrates with active surface capable of specific binding and simultaneously eliciting biomimetic mineralization would be highly superior in their performance in vivo. Molecular modelling of such organic-inorganic interface with enhanced efficiency should also satisfy geometric, stereochemical and electrostatic requirements in addition to the biospecific interaction. In the case of bone implants, surface functionalities that facilitate biomimetic mineralization are greatly favoured due to the significant amount of the inorganic content inherently associated with it. The biomimetic approaches/substrates studied and the key information gathered are collectively presented in Table 1.

**Table 1:** Biomimetic approaches for biomineralization/osseointegration and key information gathered

Biomimetic Substrate/Approaches for biomineralization and osseointegration/osteogenesis		Key information	Reference
1	Synthetic hydroxyapatite (HA) and its composites	▪ Electrostatic interaction of the HAP surface with the calcium and the phosphate ions	[105]
2	Bioglass and Bioglass-calcium phosphate composites	▪ Carbonate apatite layer formation ▪ Good osteointegration both in vitro and in vivo	[124–126]
3	U HMWPE, Biodegradable starch/ethylene vinyl alcohol blend, PU foams	▪ Formation of continuous and adherent Ca*-*P ▪ layer on the surface ▪ Needle-like crystals formed *cauliflower* like morphology	[110]
4	Surface functionalization by phosphorylation ▪ Bamboo [117] ▪ Chitosan [118] ▪ Poly (HEMA-co-MMA) [119] ▪ PVA [121] ▪ Regenerated Cellulose [122]	▪ increased number of nuclear sites and apatite formation ▪ Nucleation and porous HAP coating ▪ Direct bone bonding and elicited new bone formation ▪ promotes in vitro biomineralization and in vitro cell adhesion ▪ Increased surface roughness and leads to better binding of Calcium ions	[117] [118] [119] [121] [122]
5	▪ Surface modification Polyethersulphone ▪ PMMA (ATP coupling) Electrospun PCL - gelatin	▪ Promotes nucleation and growth of calcium phosphate ▪ Uniform apatite layer formation upto 20 μm thickness	[111, 112] [120] [140, 141]
6	RGD and BMP integrated polymer matrices	▪ Structural integrity modulation and aligned biomineralization ▪ Enhance bone specific marker protein expression and thereby mineralization	[65] [127]
6	Biodegradable Polymer Composites ▪ Viscose cellulose sponge ▪ Starch/ethylene vinyl alcohol blend (SEVA-C) ▪ Gelatin-poly(acrylic acid) matrix ▪ poly(lactide-co-glycolide)	▪ Compatible for tissue in-growth ▪ Attractive as scaffold for bone tissue engineering ▪ Promotes cell adhesion ▪ Feasibility of orientation by stretching	[143–146]
7	▪ Titanium metal ▪ Polished and gritted Titanium (Ti6Al4V)	▪ NaOH and heat treatment generates amorphous sodium titanate on the metal and induces bonelike apatite layer	[114, 115]
8	Stem Cell based approaches	▪ In vivo osteogenesis ▪ Promising source for bone tissue engineering	[132–136] [142]

## Biomimetic mineralization

A ‘biocompatible material’ invokes an appropriate host tissue responses, upon specific applications and surface modification is recognized as a successful approach to modulate cellular interactions and can be formulated to meet the requirements without altering inherent bulk functional properties [93]. Preferred biological responses and functionalities can be therefore accomplished by smart modifications of polymers by physico-chemical or biochemical ways [94–97]. ‘Biomimetic mineralization’ a process of ‘mimicking biomineralization conditions under laboratory conditions by synthetic approaches is usually accomplished with the aid of organic templates like macromolecular frameworks, cell walls or lipid membranes through specific or selective interaction between the organic moieties and the precursors of the biomineral. Approaches that facilitate biomimetic hydroxyapatite formation are extensively investigated in the last couple of decades [98–100].

### Calcium phosphate coatings

Hard tissues formation, remineralization and dissolution are complex processes involving multiple calcium phosphate phases [101] and several biological mineralization processes are associated with the formation of meta-stable intermediates which undergo subsequent transformation into better stable thermodynamic phases [102]. Kinetic studies exemplify formation of calcium phosphate precursor phases such as dicalcium phosphate dihydrate and octacalcium phosphate which eventually transforms into stable hydroxyapatite [103]. Furthermore, the nature of phases formed depends upon the pH and the type of mineralization (normal or pathological) [104]. In addition, presence of extra-lattice ions or external molecules in the system also distinctly influences the rate of mineralization and demineralization.

Kim et al. proposed that formation of bone-like apatite or calcium-rich amorphous calcium phosphate (ACP) in the in vitro environment occurs via formation of calcium-poor ACP in the early soaking period [105]. The synthesis/post-synthesis factors have detrimental roles on the functional properties of biomimetic apatites formed [106] and hence knowledge on the cellular and molecular interactions with bioceramic surfaces of impart information on the strategic design of better functioning bioceramic materials by minimizing unwanted biological effects like prolonged macrophage activation [107]. Organoapatites, that integrally incorporate amino acids like poly(L-lysine), poly(L-sodium glutamate), poly(sodium acrylate) or poly(L-lysine) have exhibited apposition of bone after 35 weeks of implantation in canine and cortical bone [108, 109].

**Fig. 3:**
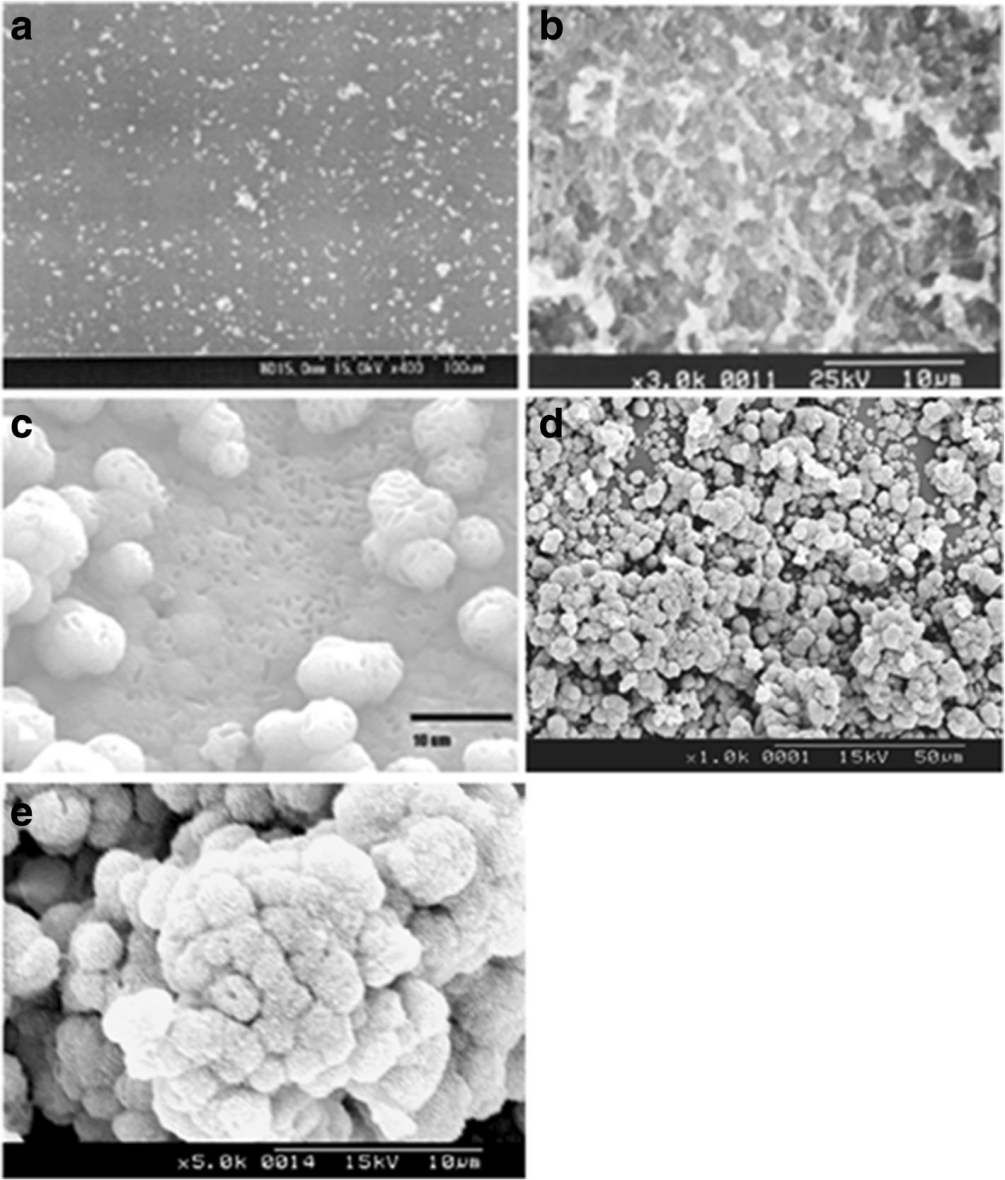
Biomimetic apatite coating formed on surface functionalized polymeric substrates (**a**): poly(methyl methacrylate) (ref: 121, with permission from Elsevier); (**b**):chitosan (ref: 120; with permission from Elsevier); (**c**): poly(vinyl alcohol) with permission from Elsevier (ref: 122); (**d**): poly(hydroxy ethyl methacrylate-co-methyl methacrylate); (**e**): high magnification image of (**d**)

Bone being an organic-inorganic hybrid tissue with 58 % mineralized part as hydroxyapatite, significant research investigations were performed to understand prominent influence of surface modification that facilitate biomimetic mineralization of calcium phosphate by graft copolymerization, plasma gas discharge, ionizing radiation, chemical derivatization, photochemical grafting, chemical modification [110–116]. Among these, surface phosphorylation has been identified as an effective method for surface functionalization [117–119]. Varma et al. demonstrated formation of calcium phosphate coating on chitosan by direct phosphorylation while PMMA required surface functionalization by coupling with ATP molecule elicit HAP coating [120]. Surface phosphorylated poly(vinyl alcohol), PVA exhibited enhanced cytocompatibility in vitro in addition to substantial apatite coating [121]. Instead of urea-phosphoric acid method, Li et al. [117] employed sodium hydroxide-phosphoric acid for phosphorylating bamboo while Granja et al. [122] phosphorylated regenerated cellulose with the aid of phosphoric acid and triethyl phosphate. In another study, the authors presented an alternative way for surface phosphorylation illustrated with poly (hydroxyl ethyl methacrylate-co methyl methacrylate) for biomimetic growth of calcium phosphate [119], and the functionalized material was demonstrated to direct bone bonding and elicited new bone formation [118]. Diverse growth morphology could be accomplished for the biomimetically grown hydroxyapatite as shown in Fig. 3(a-d). Figure 3e illustrates flower-like morphology of hydroxyapatite crystals grown biomimetically on the surface of phosphorylated poly(HEMA-co-MMA). (Biomimetic mineralization conditions are provided in the ‘Materials and Methods’ section).

### Bio-glass coating

Bioactive glass coating, another accepted method to establish calcium phosphate coating is primarily driven by the increase in the ionic product of apatite in the surrounding body fluid caused by the dissolution of Ca ^2+^ ions from the bioactive glass, which is already supersaturated with respect to apatite. The mechanism of this process is illustrated by Kokubo in several of his pioneering works [123−126]. Several investigations testify the prominent role of bioglass in favouring bone-like apatite formation elicited by large number of apatite nuclei is generated on the surfaces of bioactive glasses, triggered by the hydrated silica formed on the bioglass.

### Biomineralization through bioactive molecules

Immobilization of biomacromolecules on organic substrates by spacer groups both temporary and permanent ways has been also recognized as a bio-inspired inspiring approach as it offers greater steric freedom and hence better specific activity. Surface functional groups like -COOH, −PO_4_, −NH_2_, –OH induce site-directed or template directed nucleation and growth of hydroxyapatite. Surface functionalized biomaterial substrates with reactive anionic functional groups like -OH,-NH_2_, −COOH and -PO_4_ can persuade interactions with mineral precursor ions and thereby induce nucleation of the calcium phosphate phase under physiological as well as simulated physiological conditions. In addition to bioinspired mineralization, surface functionalization also imparts properties such as hydrophilicity, biomolecular recognition and enhanced cytocompatibility. The growth rate of apatite crystals on functionalized self-assembled monolayers on gold surface decreased in the order PO_4_ > COOH > > CONH_2_ > OH > NH_2_ > > CH_3_ [112]. Lowering of the activation energy is the driving force for the nucleation inorganic crystallization on organic surface. Concave surface accumulates an increased spatial charge concentration of the functional groups when compared to convex or planar surfaces and hence assumed as surface charge concentrated pockets leading to accelerated crystal nucleation [1].

Specific control of nucleation and growth of hydroxyapatite and better cell adhesion has been achieved by Arg-Gly-Asp (RGD) terminated self-assembled surfactant architecture comprising cystenic amino acids and phosphoserine molecule, a highly phosphorylated interface, which promoted HAP nucleation while RGD units enhanced cell adhesion [65]. Lu et al. demonstrated mineralization of rabbit skeletal muscle and increased bone marker expression with the aid of BMP-7 delivered from PLGA matrix [127].

## Contemporary strategies in bone tissue engineering

Bone is unique with respect to its organic-inorganic hybrid structure (Ca_10_(PO_4_)_6_(OH)_2_ as the inorganic phase), superior hierarchy and continuous remodelling potential [128, 129]. The composition of organic matrix of bone tissue is basically 95 % type I collagen, 5 % proteoglycans, the high molecular weight complexes of proteins and polysaccharides (e.g.: glycosaminoglycan) and non-collageneous proteins [129]. Based on the packing density, bone tissue is classified as compact (dense) and cancellous or spongy (trabecular) and continuous remodelling of bone is sequential coordinated by activities of osteoclasts, osteo-progenitor cells, osteoblasts and finally osteocytes [65]. Bone tissue engineering acquires its significance particularly due to the remodelling potential of bone. A clinically successful bone implant needs to achieve a stable interface with host tissue at the same time a good matching of the mechanical properties between the implant and the host tissue [130]. The main objective associated with bioengineering of functional bone implants is to stimulate a positive cell-material interaction followed by preferential adhesion of bone cells, synthesis of extracellular matrix and its mineralization. This biomimetic concept is achieved through biomolecular or surface functional group recognition, by surface or bulk modification of biomaterials. An osteoinductive implant promotes bone formation by causing the cells to differentiate into chondrocytes and osteoblasts. The surface of an implant can affect the cell phenotype, since a small variation in the surface charge itself can influence the cell spreading pattern.

**Fig. 4:**
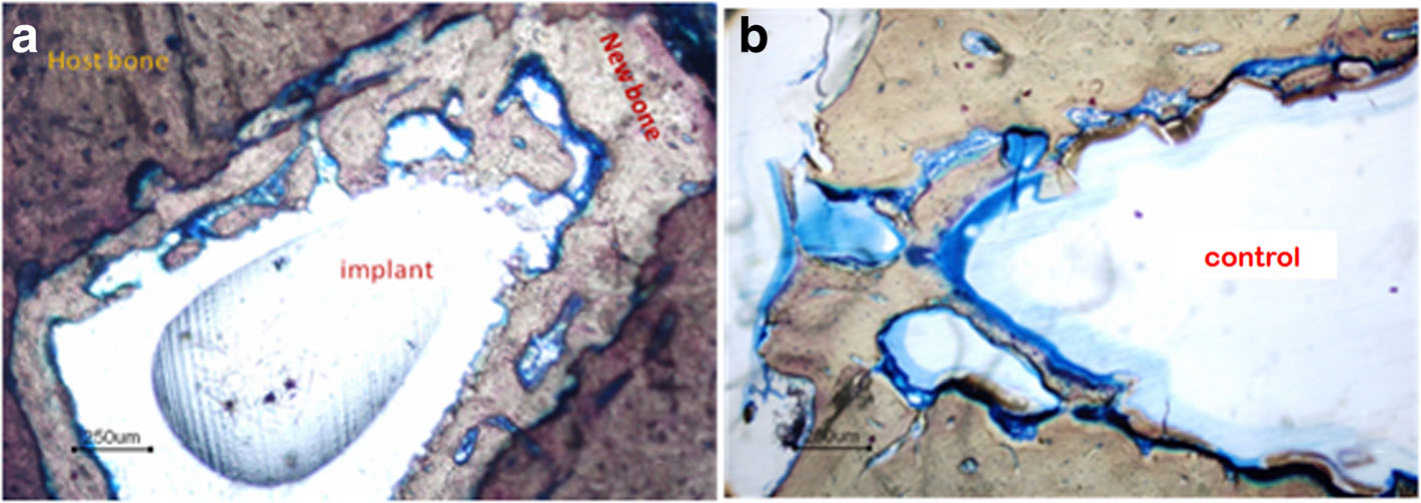
**a**: The new bone formation at surface phosphorylated poly(HEMA-co-MMA)-host bone interface (implanted in rabbit for 12 weeks) (**b**): poly methyl methacrylate-host bone interface (control) (poly(methyl methacrylate) dissolves in its monomer during embedding process)

The chemical and biochemical stimuli responsive surfaces present intriguing possibilities towards development of novel functional implant material. Biomimietic principles could be explored for regulating molecular recognition at the organic-inorganic interfaces by proper regulation of the transport processes from the extracellular fluids to the biomaterial surface. Development of multifunctional scaffolds has been widely accepted as one of the successful techniques for functional tissue engineering. Direct bonding between the implant and the host bone can be achieved through reactive surface functional groups that could ultimately facilitate biomimetic mineralization and bone regeneration in vivo. The surface phosphorylated poly(HEMA-co-MMA) promoted formation of new bone in vivo when implanted in rabbits for 12 weeks [118]. Figure 4 demonstrates that surface phosphorylated poly(HEMA-co-MMA) could elicit significant bone regeneration after 12 weeks of implantation in rabbits. (Details of implantation procedure followed are provided in the ‘Materials and Methods’ section).

In the last couple of decades, stem cells have been recognized to play potential role towards in vivo bone regeneration [131–133] though the mechanism associated with stem cell based healing and the atrophic factors associated with the profound interaction between the implanted cell type and the in vivo environment [134]. Recent attempts for regenerative orthopaedic treatment modalities propose significance of stem cell based approaches [135, 136] as well as nanotechnological approaches [137] particularly through the combination of specific cell sources, biomimetically modified scaffolds, by designing suitable bioreactor and incorporating the ideal growth factors. It is also important to mention that though there is a potential growth in the diverse yet symbiotic tissue engineering approaches, the clinical translation of these innovative approaches are relatively slower than expected.

Clinical success of bone grafts requires realization of the concept presented in “Diamond theory” of Giannoudis: that is osteogenic cells and vascularization, mechanical stability, growth factors, osteoconductive scaffolds (in combination with growths factors) [138]. The significant challenges in developing clinically successful bone tissue engineering graft is unavailability porous scaffolds that are mechanically strong enough with good vascularization potential. Contemporary grafts are limited to peripheral tissue in-growth and are also limited with currently used animal models in addition to long-time expensive validation protocols for alternative grafts [139].

Intriguing views on biomineralized porous scaffolds with 3D interconnected structures throw lights on the less explored, but highly imperative options. Porous scaffolds possessing sufficiently good mechanical properties that undergo slow degradation kinetics have a predominant role as tissue engineering constructs [140–142]. Even though resource substrates employed for this purpose are inherently biocompatible and biodegradable, most of them lack intrinsic bioactivity, typical example is cellulose [143–148]. These constructs can be transformed into effective osteocondictive platform by appropriate surface functionalization to invoke biomimetic mineralization of hydroxyapatite so that they will serve as better alternatives for consistent bone regeneration due to inherited bone-bonding potential and also function a good support for cells and exogenous factors.

Understanding the diversity of biomineraization process presents opportunities for better designing of advanced functional architectures with hierarchical porosity and organization profile that mimic the ubiquitous properties in the biological milieu. Recent developments in bioinspired biomineralization in template architectures designed with metal organic frameworks (MOF)s is a very promising and effective advancement in this area [149, 150]. Bisphosphonates and zoledronate (Zol), a third-generation bisphosphonate are evolved as pharmacology agents to treat bone disorders like osteoclast-mediated bone loss due to osteoporosis, Paget’s disease of bone, metastasis to the bone, and malignancy-associated hypercalcemia.

It is worth anticipating that the stem cell interaction with the extracellular matrix could be explored as a favourable element to initiate the desired response at the biomaterial-host tissue interface even though the specific regulatory mechanisms are only partially understood. In addition, challenges like long term immunological threats are to be addressed properly. However it could be expected that these present limiting factors will be resolved in the nearby future and implants for clinical augmentation could be derived through the multifaceted bio-inspired approaches and well designed combinations.

## Conclusions

Advances in the knowledge on cell-material interaction has imparted revolutionary ideas in designing novel smart interfaces that can facilitate the preferred signalling cascades between living cells and the implants. Precision synthesis of such advanced biomaterials with highly specific spatial distribution of bioactive sites and desired topographical architecture to invoke distinct cellular response could be made based on the comprehensive awareness of interface properties and intercellular signalling sequences. Advanced strategies employing such receptor mediated cellular responses with intrinsic biospecificity are greatly awaited by the scientific community for long-term functioning of biomimetic implants. Clinical success of these new generation smart biomaterials, despite of having all the positive advances are anticipated milestones in the near future. More quantitative approaches are welcomed for close mimicking of the advanced biofunctional implants having better clinical success. It is expected that better understanding of organic-inorganic interface will pave the way to success in this endeavour.

## Materials and methods

Hydroxyethyl methacrylate (HEMA) assay: 98 %, methyl methacrylate (MMA) assay: 99 %, and phosphorous pentoxide (assay: 98 %) were procured from Sigma-Aldrich Co. Inc. ethylene glycol dimethacrylate (assay: 98 %) from Fluka AG (Buchs, Switzerland), benzoyl peroxide (assay: 98 %) from S.D. Fine India Ltd. (Mumbai, India). All other chemicals were purchased from Ranbaxy India Pvt. Ltd. (Mumbai, India). MMA was made free of inhibitor by treating with 4 % sodium hydroxide solution for three times, followed by washing with DI water, dried by placing over anhydrous magnesium sulfate. HEMA was made free of inhibitor by passing through an inhibitor remover column (Sigma-Aldrich Co. Inc.).

Biomimetic mineralization of surface phosphorylated Poly (HEMA-co-MMA) was performed in accordance to our previously reported procedures Ref [118–120]. Short-term bone implantation was performed as per ISO 10993–6; 1994 (E) Test for local effects after implantation, clause 6.0: Test method for implantation in bone in adult Albino rabbits, from the institute animal house, Sree Chitra Tirunal Institute for Medical Sciences and Technology, Thiruvananthapuram. All implantation procedures were carried out following the rules of the Animal Ethics Committee of the institute and ISO protocol [**Reference number: B-2072005 V**]. The results presented in Figs. 3e and 4 are not published before.

## Abbreviations

ECM: Extracellular matrix
HAP: Hydroxyapatite
RGD: Arginylglycylaspartic acid
poly(HEMA-co-MMA): poly(hydroxyethyl methacrylate co methyl methacrylate)
MOF: Metal Organic Framework
MAPK: mitogen-activated protein kinase
BMP: bone morphogenetic protein
